# Brg1 coordinates multiple processes during retinogenesis and is a tumor suppressor in retinoblastoma

**DOI:** 10.1242/dev.124800

**Published:** 2015-12-01

**Authors:** Issam Aldiri, Itsuki Ajioka, Beisi Xu, Jiakun Zhang, Xiang Chen, Claudia Benavente, David Finkelstein, Dianna Johnson, Jennifer Akiyama, Len A. Pennacchio, Michael A. Dyer

**Affiliations:** 1 Department of Developmental Neurobiology, St. Jude Children's Research Hospital, Memphis, TN 38105, USA; 2 Center for Brain Integration Research (CBIR), Tokyo Medical and Dental University (TMDU), Tokyo 113-8510, Japan; 3 Department of Computational Biology, St. Jude Children's Research Hospital, Memphis, TN 38105, USA; 4 Department of Ophthalmology, University of Tennessee Health Science Center, Memphis, TN 38163, USA; 5 Lawrence Berkeley National Laboratory, Genomics Division, Berkeley, CA 94701, USA; 6 Department of Energy, Joint Genome Institute, Walnut Creek, CA 94598, USA; 7 Howard Hughes Medical Institute, Chevy Chase, MD 20815, USA

**Keywords:** SWI/SNF, Epigenetics, Retina development, Retinoblastoma, Mouse

## Abstract

Retinal development requires precise temporal and spatial coordination of cell cycle exit, cell fate specification, cell migration and differentiation. When this process is disrupted, retinoblastoma, a developmental tumor of the retina, can form. Epigenetic modulators are central to precisely coordinating developmental events, and many epigenetic processes have been implicated in cancer. Studying epigenetic mechanisms in development is challenging because they often regulate multiple cellular processes; therefore, elucidating the primary molecular mechanisms involved can be difficult. Here we explore the role of Brg1 (Smarca4) in retinal development and retinoblastoma in mice using molecular and cellular approaches. Brg1 was found to regulate retinal size by controlling cell cycle length, cell cycle exit and cell survival during development. Brg1 was not required for cell fate specification but was required for photoreceptor differentiation and cell adhesion/polarity programs that contribute to proper retinal lamination during development. The combination of defective cell differentiation and lamination led to retinal degeneration in *Brg1*-deficient retinae. Despite the hypocellularity, premature cell cycle exit, increased cell death and extended cell cycle length, retinal progenitor cells persisted in *Brg1*-deficient retinae, making them more susceptible to retinoblastoma. ChIP-Seq analysis suggests that Brg1 might regulate gene expression through multiple mechanisms.

## Abstract (precis)

**Summary:** The SWI/SNF protein Brg1 controls cell cycle length, cell cycle exit and cell survival, and is required for cell differentiation and retinal lamination, in the developing mouse retina.

## INTRODUCTION

Multipotent retinal progenitor cells undergo unidirectional changes in competence during development to produce each of the seven classes of cell types in an evolutionarily conserved birth order (Cepko et al., 1996; Livesey and Cepko, 2001). Proliferation must be precisely controlled during this process to ensure that each cell type is produced at the correct time during development and in the appropriate proportion (Dyer and Cepko, 2001a). When proliferation and differentiation become uncoupled during retinogenesis, a developmental tumor of the retina called retinoblastoma can form (Dyer and Bremner, 2005). The initiating genetic lesion in retinoblastoma is *RB1* gene inactivation (Friend et al., 1986; Knudson, 1971). RB1 controls proliferation, cell survival and differentiation during the development of the retina and many other tissues (Donovan and Dyer, 2004; Zhang et al., 2004; Donovan et al., 2006; Johnson et al., 2006, 2007; Sun et al., 2006; Dyer, 2007; McEvoy et al., 2011). Therefore, the Rb pathway lies at the heart of the regulatory network that coordinates the balance between proliferation and differentiation during retinal development. The precise mechanism by which RB1 coordinates these different processes in multipotent retinal progenitor cells is unknown.

RB1 and the other two Rb family members [P107 (RBL1) and P130 (RBL2)] can directly regulate transcription by binding E2Fs at their cognate promoters (Ishida et al., 2001; Muller et al., 2001; Ren et al., 2002; Weinmann et al., 2001; Cam and Dynlacht, 2003; Wells et al., 2002; Iwanaga et al., 2006). However, the Rb family of proteins may coordinate retinal progenitor cell proliferation and differentiation through direct or indirect epigenetic processes. Indeed, RB1 has been implicated in regulating most major epigenetic processes, including miRNA expression, DNA methylation, histone modification and ATP-dependent chromatin reorganization (Chi et al., 2010; Lu et al., 2007; Benetti et al., 2008; Wen et al., 2008; Bourgo et al., 2009; Gonzalo and Blasco, 2005). Recent studies suggest that RB1 inactivation leads to epigenetic changes in key cancer and differentiation pathways in the developing retina (McEvoy et al., 2011; Zhang et al., 2012).

To study the mechanism of RB1-mediated epigenetic regulation of cell proliferation, differentiation and survival, we have focused on BRG1 (SMARCA4), which is an ATPase subunit of the SWI/SNF complex involved in nucleosome mobilization during development and tumorigenesis (Dunaief et al., 1994). BRG1 can bind all three Rb family members (Dunaief et al., 1994), and genetic analysis of human tumors has suggested that *BRG1* is a tumor suppressor (Reisman et al., 2009; Medina et al., 2008; Rodriguez-Nieto et al., 2011; Hargreaves and Crabtree, 2011). For example, it was reported that a subgroup of patients with childhood medulloblastomas had recurrent mutations in *BRG1* (Robinson et al., 2012). In addition, *Brg1* heterozygous mice are prone to forming epithelial tumors, and several types of lung cancer cell lines exhibit frequent inactivating mutations in *BRG1* (Dunaief et al., 1994; Medina et al., 2008).

Importantly, BRG1 has been linked to progenitor cell proliferation, differentiation and survival in a variety of organs (e.g. the heart), the central nervous system and T cells (Hang et al., 2010; Matsumoto et al., 2006; Wurster and Pazin, 2008). For example, deletion of *Brg1* in embryonic mouse cardiomyocytes contributes to heart defects that cause embryonic lethality (Hang et al., 2010). The myocardium in *Brg1*-deficient embryos is thin and compact, yet there was no evidence of increased cell death. Instead, reduced proliferation and premature differentiation of *Brg1*-deficient cardiomyocytes suggest that Brg1 is required to maintain these cells in an immature state (Hang et al., 2010). Similarly, in the developing mouse cortex, inactivation of *Brg1* leads to a dramatic reduction in tissue size (Matsumoto et al., 2006). The pool of proliferating progenitor cells is rapidly depleted as the cells prematurely differentiate. However, in contrast to the developing heart, at least a subset of *Brg1*-deficient neurons die shortly after they exit the cell cycle, suggesting that Brg1 plays a role in maintaining the pool of progenitor cells and promoting survival of a subset of differentiated neurons (Matsumoto et al., 2006).

Brg1 and other BAF-associated proteins are expressed in murine retinal progenitor cells and differentiated retinae (Lamba et al., 2008). Here, we explore the role of Brg1 in murine retinal development and retinoblastoma. We found that when Brg1 was inactivated in the developing mouse retina, there was a significant reduction in retinal and eye size and a dramatic disruption in retinal lamination. The microphthalmia in *Brg1*-deficient retinae was caused by a combination of cell death and lengthening of the cell cycle. Retinal cell fate specification was normal in *Brg1*-deficient retinae, but defects in rod and cone photoreceptor differentiation led to retinal degeneration. We demonstrate that genes involved in cell proliferation, adhesion, polarity and photoreceptor differentiation are direct targets of Brg1 in the developing retina. In addition, we show that despite the increased cell death and cell cycle length caused by *Brg1* deficiency, Brg1 is a tumor suppressor in retinoblastoma.

## RESULTS

### *Brg1*-deficient retinae are hypocellular

**Fig. 1.**
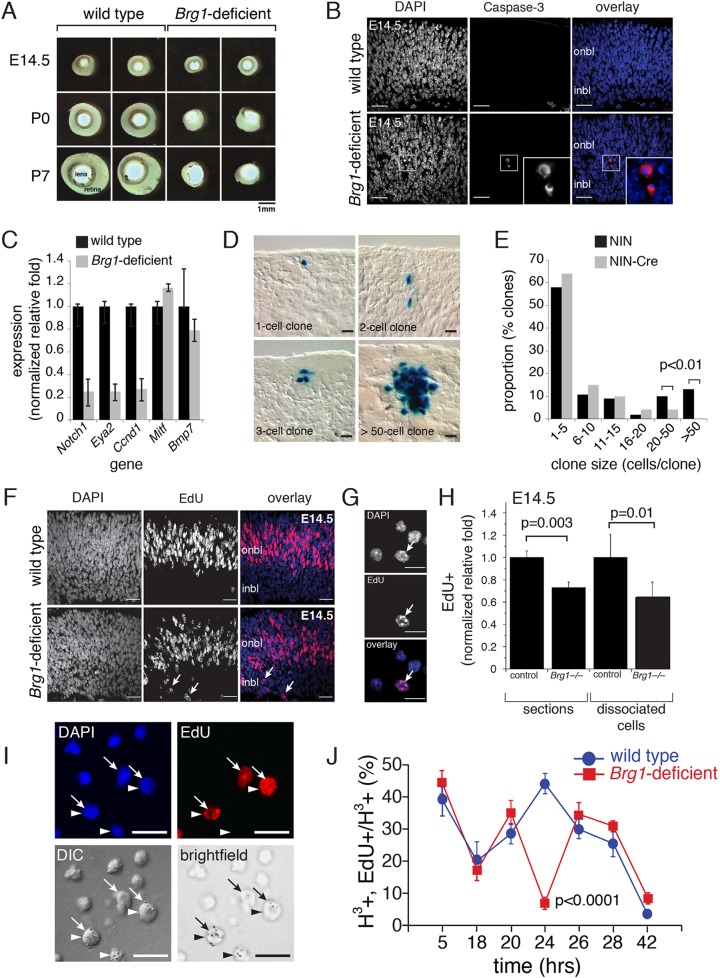
**Brg1-deficient retinae are hypocellular.** (A) Isolated mouse retinae with lens. (B) Micrographs of activated caspase 3 immunofluorescence (red) of E14.5 retinal cryosections, with DAPI nuclear counterstaining (blue). (C) Quantitative real-time PCR analysis using TaqMan probes for E14.5 retinae. Each bar is the mean and s.d. of replicate PCR from triplicate samples. (D) Retinal cryosections stained with X-gal showing clones of cells derived from single retinal progenitor cells infected with a replication-incompetent retrovirus expressing nuclear β-galactosidase. (E) The proportion of clones for each clone size for wild-type (NIN) and Brg1-deficient (NIN-Cre) retinae. (F) EdU staining (red) of E14.5 retinal cryosections, with DAPI nuclear counterstaining (blue). (G) Dissociated cells stained with EdU (red) and DAPI (blue). (H) The proportion of EdU^+^ cells from retinal sections and dissociated cell scoring at E14.5. (I) EdU staining (red) with DAPI nuclear countertstain (blue) and detection of [^3^H]-thymidine in overlaid autoradiographic emulsion (DIC and brightfield images). Arrows indicate EdU^+^ cells and arrowheads indicate [^3^H]-thymidine^+^ cells. (J) The proportion of EdU^+^ cells among [^3^H]-thymidine^+^ cells at each time point for wild-type and Brg1-deficient retinae. DIC, differential interference contrast. Scale bars: 25 µm in B,D,F; 10 µm in G,I.

To explore the role of *Brg1* in retinal development, we generated *Chx10-Cre;Brg1^Lox/Lox^* mice (*Chx10* is also known as *Vsx2*). The *Chx10-Cre* transgene was expressed in retinal progenitor cells throughout development in a mosaic pattern, providing adjacent wild-type and *Brg1*-deficient stripes that spanned all three cellular layers of the retina (Rowan and Cepko, 2004) (Fig. S1). In postnatal day (P) 12 *Chx10-Cre;Brg1^Lox/Lox^;Rosa-YFP* retinae, 89±4% of GFP^+^ cells that had undergone Cre-mediated recombination lacked Brg1 protein as visualized by immunofluorescence (Fig. S1). At embryonic day (E) 14.5, the retinae in the *Chx10-Cre;Brg1^Lox/Lox^* mice were slightly smaller than those in *Chx10-Cre;Brg1^Lox/+^* or *Chx10-Cre;Brg1^+/+^* littermates (Fig. 1A; data not shown). This reduced size was more pronounced at P0 and P4 (Fig. 1A). To determine whether the reduced eye and retinal sizes were due to increased apoptosis, we immunostained E14.5, P0 and P4 retinal cryosections with an antibody specific for activated caspase-3. Scoring the proportion of activated caspase-3^+^ cells, we found an increase in the *Chx10-Cre;Brg1^Lox/Lox^* retinae at all stages (Fig. 1B; data not shown). However, statistical significance was achieved only in P4 retinae (0.1±0.2% for control and 0.6±0.2% for *Brg1* deficient; *P*=0.01). To test whether p53 (Trp53) contributed to the increase in apoptosis in *Brg1*-deficient retina, we generated *Chx10-Cre;Brg1^Lox/Lox^;p53^Lox/Lox^* mice*.* The eye size and level of apoptosis in these retinae were indistinguishable from those of *Chx10-Cre;Brg1^Lox/Lox^* retinae (data not shown).

Next, we analyzed the expression of *Ccnd1*, *Notch1* and *Eya1* by qPCR using TaqMan probes. All three of these retinal progenitor cell genes were significantly reduced in the *Brg1-*deficient retinae at E14.5, but *Mitf* (retinal pigment epithelium) and *Bmp7* (ciliary body, iris) were not (Fig. 1C). To analyze the clonal expansion of individual retinal progenitor cells during retinogenesis, we indelibly marked single retinal progenitor cells by infecting E14.5 retinal explants with replication-incompetent retroviruses. Each clone derived from an individual retinal progenitor cell was reconstructed from serial sections, and the number of cells per clone was scored as described previously (Fig. 1D) (Zhang et al., 2004; Dyer and Cepko, 2000; Dyer et al., 2003). The proportion of large clones (>20 cells per clone) derived from retinal progenitor cells lacking *Brg1* was significantly reduced (*P*<0.01; Fig. 1E).

The results from the clonal analysis combined with the reduced retinal size suggested that either the cell cycle exit was premature or the cell cycle was lengthened in the absence of *Brg1*. To measure the proportion of cells in S-phase in wild-type retinae and *Brg1*-deficient retinae, we performed a 1-h EdU pulse-labeling experiment at E14.5 and calculated the proportion of EdU^+^ cells in retinal cryosections and dissociated cells. The proportion of EdU^+^ cells was significantly less in the *Brg1-*deficient retinae (*P*=0.003 for retinal sections and *P*=0.01 for dissociated cells; Fig. 1F-H).

To test whether the overall cell cycle length increased in retinal progenitor cells from *Brg1*-deficient retinae, we performed a double-labeling experiment with P0 wild-type and *Brg1*-deficient retinae. Retinae were pulsed with [^3^H]-thymidine for 1 h. Then, at several time points over 42 h, the retinae were labeled for 1 h with EdU. The tissue was dissociated, plated on glass slides, immunostained for EdU, overlaid with autoradiographic emulsion to detect the [^3^H]-thymidine, and processed (Fig. 1I). We scored the proportion of Edu^+^ among [^3^H]-thymidine^+^ cells at each time point to monitor the progression of retinal progenitor cells through the cell cycle. Twenty-four hours after the initial [^3^H]-thymidine labeling, the proportion of cells that were positive for both EdU and [^3^H]-thymidine was significantly reduced (*P*<0.0001) in the *Brg1*-deficient retinae (Fig. 1J). These data are consistent with previous estimates of 16 to 24 h for murine retinal progenitor cell cycle length at P0 (Alexiades and Cepko, 1996). However, by 26 h, the *Brg1*-deficient retinal progenitor cells entered a second round of S-phase, similar to their wild-type counterparts. More importantly, these data suggest that cell cycle progression is delayed in the absence of Brg1.

### Brg1 is required for photoreceptor differentiation

**Fig. 2.**
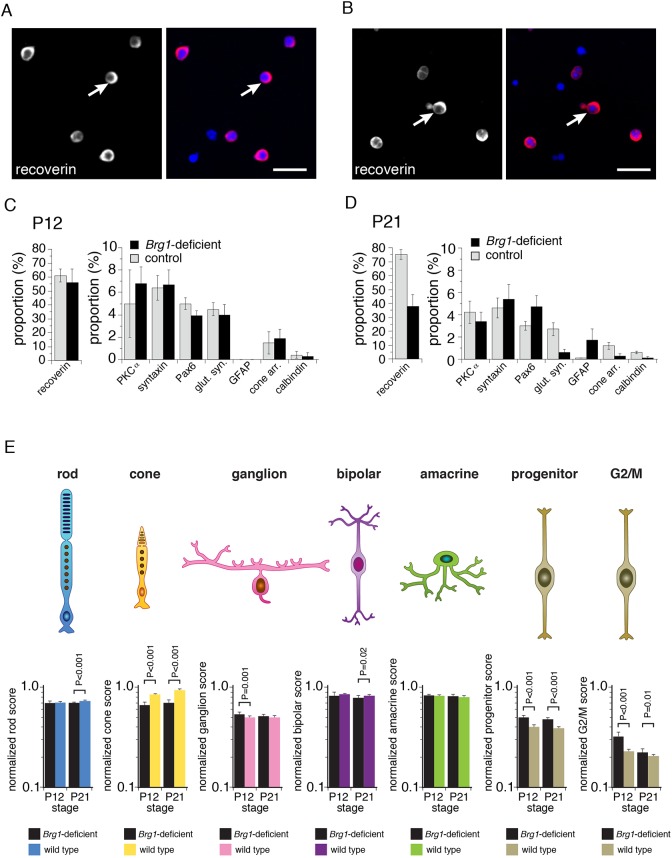
**Cell fate specification and differentiation in Brg1-deficient retinae.** (A,B) Dissociated cells immunostained for recoverin expression (red) with DAPI nuclear counterstain (blue) in P12 wild-type and Brg1-deficient retinae. Arrows indicate representative recoverin-immunopositive cells. (C,D) The proportion of cells immunopositive for cell type-specific markers at P12 and P21. Each bar represents the mean and s.d. of scoring of 250 cells in duplicate across triplicate samples. (E) Histograms of normalized cell type-specific gene expression signature scoring at P12 and P21 for wild-type and Brg1-deficient retinae from gene expression array analysis. Scale bars: 10 µm.

To determine whether Brg1 is required for retinal cell fate specification and/or differentiation, we scored the proportion of rods, cones, Müller glia, amacrine, horizontal, and bipolar cells in wild-type and *Brg1-*deficient retinae. Individual retinae were dissociated, plated on glass slides, and immunostained using antibodies specific for each of the retinal cell types (Fig. 2A,B; data not shown). Cells (*n*=250) were scored in duplicate for each antibody on at least three independent retinae at P12 and P21 (Fig. 2C,D). At P12, the proportion of immunopositive cells did not differ significantly (Fig. 2C). However, by P21, the proportion of cells immunopositive for markers of photoreceptors, Müller glia, and horizontal neurons was significantly reduced (*P*<0.01; Fig. 2D). These data were validated by qPCR (Fig. S2). The observed increase in Gfap^+^ Müller glia cells was consistent with reactive gliosis (Fig. 2D).

To extend these data, we isolated RNA from wild-type and *Brg1-*deficient retinae and performed gene expression array analysis (*n*=25 arrays) (Table S1). The only significantly downregulated pathways at P12 and P21 in *Brg1*-deficient retinae were related to photoreceptor differentiation and function (Table S1, Fig. S2). The photoreceptor genes were downregulated at P12 before the photoreceptors were lost at P21; thus, Brg1 may be required for photoreceptor maturation during neonatal retinogenesis.

None of the pathways was significantly upregulated at P12; however, at P21 the pathways implicated in lens development, inflammation, and the adaptive immune response were significantly upregulated (Table S1). To determine whether gene expression profiles for particular cell types were altered at P12 or P21, we used the cell type signatures of retinal progenitor cells, rod photoreceptors, cone photoreceptors, amacrine cells, ganglion cells, and Müller glia (Table S2), as described previously (McEvoy et al., 2011; Trimarchi et al., 2008; Cherry et al., 2009; Roesch et al., 2008). We also analyzed a group of previously characterized genes that regulate the G2/M-phase of the cell cycle in retinal progenitor cells to compare the proliferation signature in the wild-type and *Brg1*-deficient retinae at P12 and P21 (Table S2). The largest differences were decreases in the photoreceptor signatures at P21 and increases in the retinal progenitor and G2/M signatures at P12 (Fig. 2E, Table S3).

### *Brg1*-deficient retinae exhibit defects in retinal lamination

**Fig. 3.**
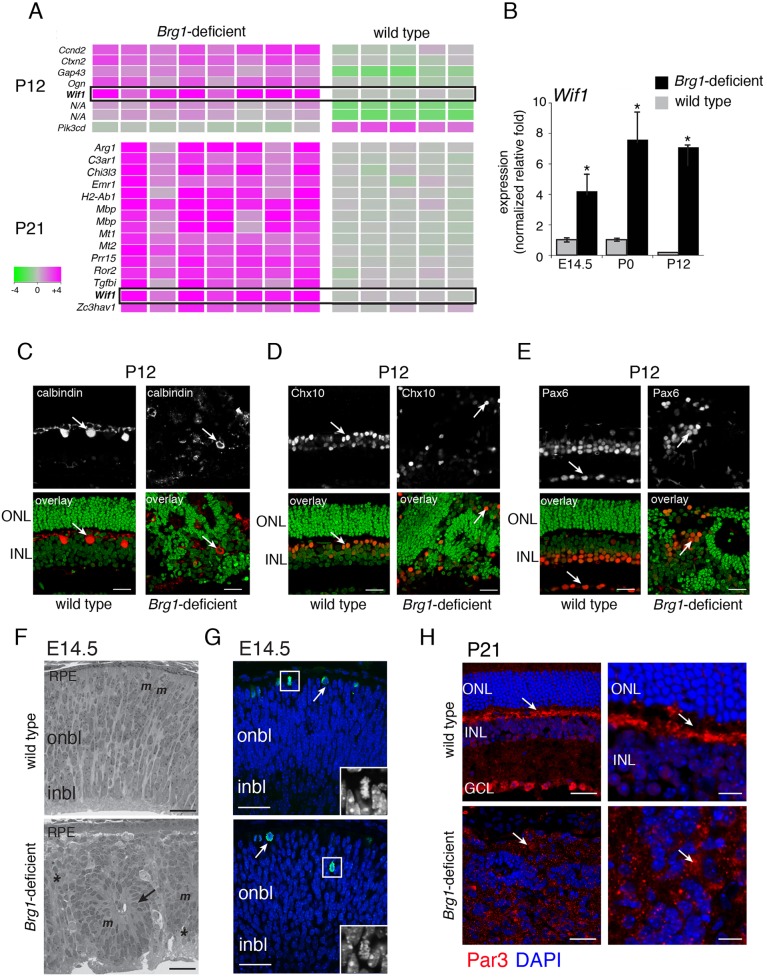
**Brg1-deficient retinae have defects in retinal lamination.** (A) Heat map of gene expression array analysis for P12 and P21 wild-type and Brg1-deficient retinae. Each column is a biological replicate. (B) Quantitative PCR analysis of *Wif1* expression using TaqMan probes. Each bar is the mean and s.d. of duplicate PCR reactions from triplicate samples. *P<0.05. (C-E) Immunofluorescent staining of calbindin, Chx10 and Pax6 (red) of P12 wild-type and Brg1-deficient retinae, with green nuclear counterstain. Arrows indicate immunopositive cells. (F) Brightfield micrograph of Toluidine Blue-stained section of wild-type and Brg1-deficient retinae. *m*, mitotic figure; asterisks, dying cells; arrow, rosette. (G) Wild-type (top) and Brg1-deficient (bottom) E14.5 retinal cryosections stained for pH3 (red) and with nuclear counterstain (blue). Arrows indicate pH3-immunopositive cells. A representative mitotic cell is magnified in the inset. (H) Wild-type and Brg1-deficient retinal sections immunostained for Par3 (red) and with DAPI (blue) at P21. Arrows indicate localization of Par3 immunofluorescence. ONL, outer nuclear layer; INL, inner nuclear layer; GCL, ganglion cell layer; onbl, outer neuroblastic layer; inbl, inner neuroblastic layer; RPE, retinal pigment epithelium. Scale bars: 25 µm.

Among the differentially expressed genes with at least a 4-fold change in gene expression, Wnt inhibitory factor 1 (*Wif1*) was the only one that was upregulated at both P12 and P21 in the *Brg1*-deficient retinae (Fig. 3A, Table S1). This increase in *Wif1* expression was validated by qPCR analysis at E14.5, P0 and P12 (Fig. 3B). Wif1 is a secreted protein that antagonizes WNT signaling by sequestering WNTs (Malinauskas et al., 2011). Importantly, the defects in eye and retinal size in *Brg1*-deficient mice were very similar to those described previously when β-catenin (*Ctnnb1*) was conditionally inactivated in the developing retina (Fu et al., 2006). In addition to hypocellularity, *Ctnnb1*-deficient retinae had defects in retinal lamination, which were consistent with perturbations in cell adhesion and/or cell polarity (Fu et al., 2006).

To determine whether *Brg1-*deficient retinae also have defects in retinal organization, we immunostained P12 retinal cryosections using antibodies against rhodopsin, recoverin, calbindin, PKC-α (Prkca), Chx10, Pax6, cone arrestin, glutamine synthetase and syntaxin. The retinal lamination of *Brg1-*deficient retinae was extensively disrupted (Fig. 3C-E; data not shown). The earliest stage we analyzed was E14.5; retinal lamination was disrupted then and throughout the subsequent developmental stages examined (Fig. 3F). During retinal development, retinal progenitor cells undergo interkinetic nuclear migration, whereby the M-phase occurs at the apical surface and the S-phase occurs at the basal surface. We immunostained E14.5 retinae using an antibody against phospho-histone H3 (pH3). This antibody labeled mitotic cells in the M-phase of the cell cycle. In wild-type embryos, the pH3^+^ cells were found in their expected position at the apical edge of the retinae, but in *Brg1*-deficient retinae some were displaced to inner regions of the outer neuroblastic layer (Fig. 3G). Additionally, we examined the integrity of the apical retina upon loss of *Brg1* by assessing the expression of a group of proteins that are associated with adherens junctions [ZO-1 (Tjp1), aPKC-λ (Prkci) and γ-tubulin] localized to that region of the retina. In *Brg1*-deficient retinae, the apical localization of ZO-1, aPKC-λ and γ-tubulin was disrupted (Fig. S3).

The defect in retinal lamination in *Ctnnb1*-deficient retinae was associated with abnormal expression and localization of N-cadherin (cadherin 2), F-actin, Par3 (Pard3) and Par6 (Pard6a) (Fu et al., 2006). Immunostaining of retinal sections from wild-type and *Brg1*-deficient retinae gave similar results (Fig. 3H, Fig. S3; data not shown). Transmission electron micrographs of E14.5 *Chx10-Cre;Brg1^Lox/Lox^* retinae revealed extensive disorganization of retinal lamination in the developing retina and the presence of dying cells in the mature retina (Figs S3-S5). The nuclei of retinal progenitor cells undergoing interkinetic nuclear migration have an elongated morphology along the apical-basal axis due to their apical-basal polarity (Fig. S2). To determine whether there was a defect in apical-basal polarity of retinal progenitor cells, we calculated the relative differences in length versus width of elongated nuclei in wild-type and *Brg1*-deficient E14.5 retinae. We traced ten representative long nuclei and ten representative round nuclei and calculated their shape factor (largest diameter/smallest diameter; Fig. S2). The elongated nuclei were approximately twice as long as they were wide. Using these criteria, we scored the proportion of elongated nuclei in two independent fields of duplicate E14.5 retinae for wild-type and *Brg1*-deficient embryos (*n*=924 nuclei). There were significantly more (*P*=0.0013) elongated nuclei in wild-type (85±4.4%) than in *Brg1*-deficient (52±11%) E14.5 retinae (Fig. S2). These data are consistent with the hypothesis that inactivation of *Brg1* leads to a defect in retinal progenitor cell polarity.

### Brg1 regulates retinal lamination through epigenetic mechanisms

**Fig. 4.**
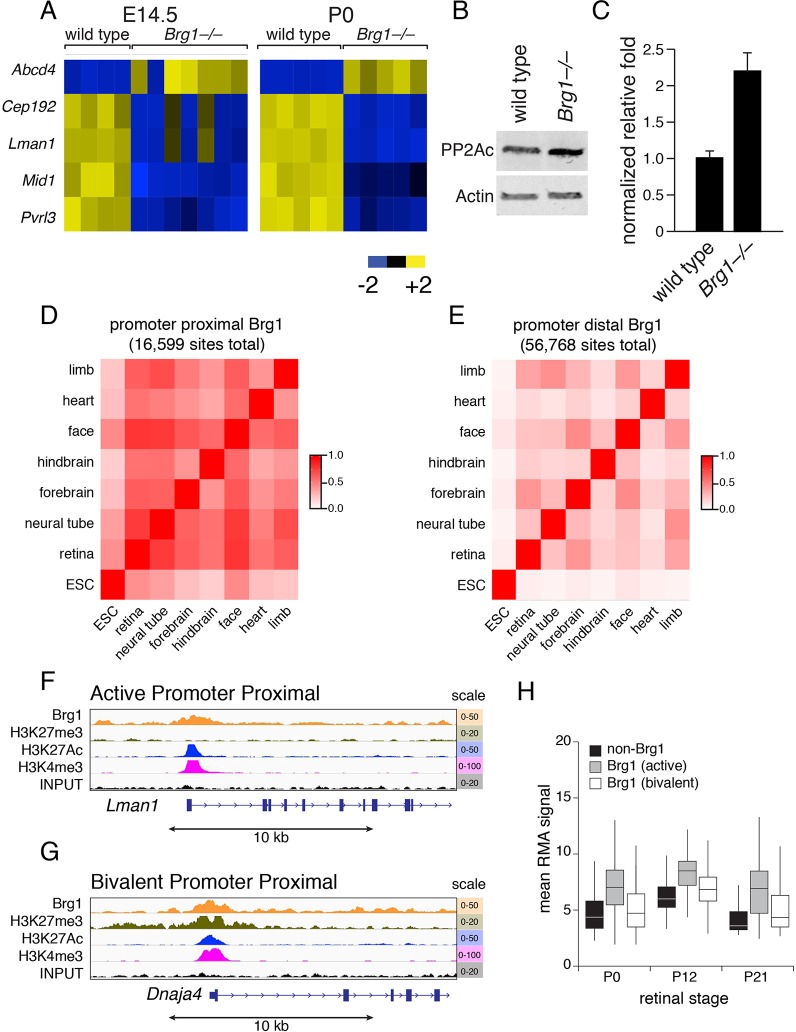
**Brg1 target genes are involved in cell polarity and cell adhesion.** (A) Heat map of gene expression array analysis of five genes dysregulated at E14.5 and P0 in Brg1-deficient retinae. Each column is a biological replicate. (B) Immunoblot of PP2Ac and β-actin expression in wild-type and Brg1 P0 retinae. Actin, loading control. (C) Quantitation of PP2Ac protein expression from duplicate samples. Each bar is the mean and s.d. of PP2Ac expression normalized to β-actin expression. (D) Heat map of correlation for promoter-proximal Brg1 ChIP peaks across different tissues. Promoter-proximal sites are within 1 kb of the transcriptional start site of a gene. (E) Heat map of correlation for promoter-distal Brg1 ChIP peaks across different tissues. (F) Representative ChIP-Seq traces for an active promoter-proximal site. (G) Representative ChIP-Seq traces for a bivalent promoter-proximal site. (H) Box plot of expression of Brg1 target genes with active or bivalent epigenetic marks at P0, P12 and P21. ESC, embryonic stem cell; RMA, robust multiarray average.

To gain additional insight into the molecular mechanism underlying retinal laminar deficiencies in *Brg1*-deficient retinae, we extended our gene expression array analysis to E14.5 and P0. Four genes (*Cep192*, *Mid1*, *Pvrl3* and *Lman1*) were downregulated in E14.5 and P0 *Brg1-*deficient relative to wild-type retinae (Fig. 4A, Table S4). *Lman1* and *Pvrl3* encode transmembrane proteins (Hao et al., 2014; Zheng et al., 2013, 2010; Takai et al., 2003a; Shingai et al., 2003; Inagaki et al., 2003; Yasumi et al., 2003; Takai and Nakanishi, 2003), while *Mid1* and *Cep192* coordinate microtubule assembly (Cainarca et al., 1999; Joukov et al., 2014; Sonnen et al., 2013; Moser et al., 2013). Lman1 is thought to regulate the selective transportation of cargo proteins, such as rhodopsin, from the endoplasmic reticulum to the Golgi apparatus in photoreceptors (Hao et al., 2014). However, retinal progenitor cell proliferation and retinal lamination are normal in *Lman1-*deficient mice (Hao et al., 2014), suggesting that downregulation of *Lman1* in *Brg1*-deficient retinae is not the major cause of the microphthalmia and retinal lamination defects in our analyses.

*Pvrl3* encodes a cell adhesion protein that is required for the formation of cell-cell junctions in neurons and other cell types (Takai et al., 2003b). Mutations in this gene are associated with congenital ocular defects in the lens and ciliary body in humans (Lachke et al., 2012). *Pvrl3*-deficient mice have microphthalmia and defects in cell adhesion between the pigmented and non-pigmented ciliary epithelial cells of the eye (Inagaki et al., 2005). Lamination is normal in the *Pvrl3*-deficient mouse retina, and it has been proposed that the microphthalmia is a secondary effect of perturbations in the formation of vitreal humor due to the defects in the ciliary epithelium (Inagaki et al., 2005). Cep192 orchestrates a signaling cascade that is required for centrosome maturation and bipolar spindle assembly during mitosis (Kim and Dynlacht, 2013). Downregulation of Cep192 in *Brg1*-deficient retinae might perturb the coordination of interkinetic nuclear migration and the timing of S-phase and M-phase in the developing retina. This is consistent with the longer cell cycle length in the *Brg1-*deficent retinal progenitor cells and the defect in apical localization of M-phase initiation.

Mutations in the *MID1* gene in humans cause X-linked Opitz/BBB syndrome, which is characterized by abnormal closure of midline structures during development (Quaderi et al., 1997). MID1 is an E3 ubiquitin ligase associated with microtubules throughout the cell cycle as part of a large multiprotein complex (Cainarca et al., 1999). In chick neural crest, Mid1 can affect cranial neural crest cell migration and matrix remodeling (Latta and Golding, 2012). One target of Mid1 is the phosphatase PP2Ac (Ppp2c), which regulates microtubule organization and tight junction formation (Kurimchak and Grana, 2012a,b; Nunbhakdi-Craig et al., 2002). Consistent with the hypothesis that Mid1 negatively regulates PP2Ac protein levels by ubiquitin-mediated proteolysis, we found that PP2Ac protein levels were elevated in the *Brg1-*deficient retinae at P0 (Fig. 4B,C).

Only one gene (*Abcd4*) was upregulated in *Brg1*-deficient retinae at both E14.5 and P0 (Fig. 4A, Table S4). Mutations in *ABCD4* can cause cobalamin C disease due to failure to release vitamin B_12_ from lysosomes (Coelho et al., 2012). Vitamin B_12_ is important for retinal homeostasis; progressive retinal degeneration has been reported in infants and young children with cobalamin C disease (Schimel and Mets, 2006).

Brg1 can bind active and repressive regulatory sequences in a tissue-specific manner (Attanasio et al., 2014). To determine whether any of the genes that are dysregulated in the *Brg1*-deficient retinae have adjacent Brg1 binding sites, we used the *Brg1-FLAG* knock-in mouse strain developed by Attanasio et al. (2014). P0 retinae from *Brg1-FLAG* pups were fixed in formaldehyde, and chromatin was prepared for anti-FLAG ChIP-Seq analysis. To relate the Brg1 binding domains and alterations in gene expression to transcriptionally active, repressed or bivalent chromatin domains in the retina, we also performed ChIP-Seq and gene expression array analysis on wild-type retina at P0, P12-14 and P21 using antibodies against H3K4me3, H3K4me1, H3K27Ac and H3K27me3.

In a previous study using a *Brg1-FLAG* knock-in mouse strain, the authors identified 55,967 Brg1 peaks across diverse tissue samples (embryonic heart, limb, hindbrain, forebrain, neural tube, face and embryonic stem cells) (Attanasio et al., 2014). In that study, among the 55,967 Brg1 peaks, 26% (14,513) were defined as promoter proximal, falling within 1 kb of an annotated RefSeq gene or transcriptional start site from the UCSC database (Attanasio et al., 2014). All other peaks were considered promoter-distal sites and many of those fell within evolutionarily conserved regulatory regions. Using the same criteria, we identified 47,902 Brg1 peaks in the P0 retinae, 34% (16,428) of which were promoter proximal. Next, we combined our retinal ChIP data with the previously published datasets and analyzed the overlap in promoter-proximal and promoter-distal sites across tissues and cell types (Fig. 4D,E, Table S5). Overall, the promoter-proximal sites were more highly conserved across tissues and the overlap in Brg1 peaks from P0 retina was highest with the E9.5 face (80%) and E9.5 neural tube (77%) (Fig. 4E, Table S5). Among the 16,428 promoter-proximal Brg1 peaks, 88% (14,468) also had overlapping H3K4me3 and H3K27Ac peaks suggesting that they are active promoters (Fig. 4F, Table S6). By contrast, only 17% (2731) of Brg1 peaks in P0 retina promoter-proximal sites have overlapping H3K4me3 and H3K27me3 consistent with bivalency (Fig. 4G, Table S7). We also analyzed the expression of the Brg1 target genes with active or bivalent epigenetic promoter-proximal marks at P0, P12 and P21 and showed a correlation between the epigenetic state and gene expression (Fig. 4H).

**Fig. 5.**
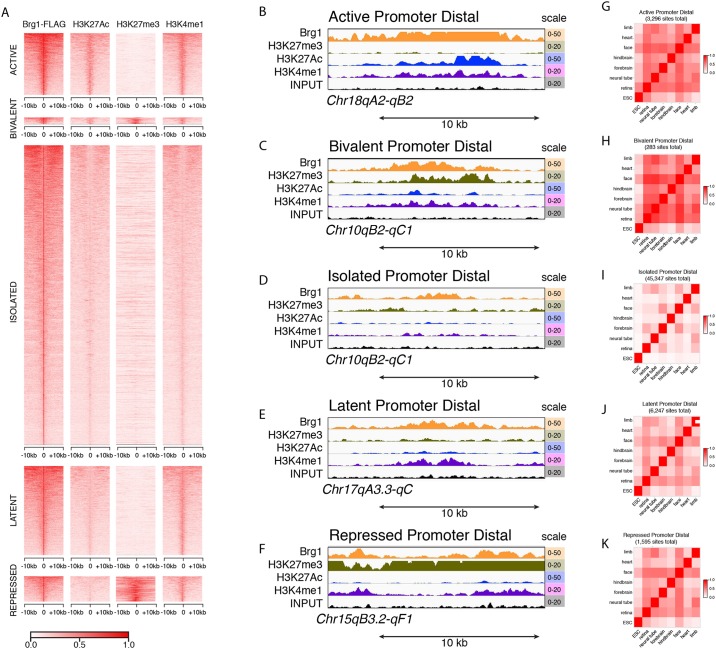
**Identification and characterization of Brg1 promoter-distal sites in P0 retinae.** (A) Each promoter-distal peak for Brg1 is represented by a horizontal line and the intensity represents the number of normalized reads at that position for Brg1, H3K27Ac, H3K27me3 or H3K4me1. All peaks are centered at 0 in the plot and span 10 kb upstream and downstream from the center of the peak. The five classes (active, bivalent, isolated, latent, repressed) of promoter-distal regions are indicated. (B-F) ChIP-Seq traces of genomic regions representative of each type of promoter-distal Brg1 binding site. (G-K) Heat map of correlation for the five types of promoter-distal Brg1 ChIP peaks across different tissues.

For the promoter-distal sites we identified five categories as defined previously (Attanasio et al., 2014). Active Brg1 sites accounted for 13% (4099) of the P0 retinal peaks and had overlapping H3K27Ac and H3K4me1 peaks (Fig. 5A,B). Bivalent Brg1 sites with overlapping H3K27me3, H3K27Ac and H3K4me1 constituted only 1% (455) of all Brg1 peaks in P0 retinae (Fig. 5C), and isolated Brg1 binding peaks with no overlap of H3K27me3, H3K27Ac or H3K4me1 comprised the largest category accounting for 61% (19,416) (Fig. 5D). Latent Brg1 sites with overlapping H3K4me1 peaks accounted for 18% (5793) of the P0 retinal peaks, while repressed Brg1 sites with overlapping H3K27me3 peaks accounted for 5% (1720) (Fig. 5E,F). The active and bivalent promoter-distal Brg1 binding sites were most similar across tissues (Fig. 5G,H, Table S8).

**Fig. 6.**
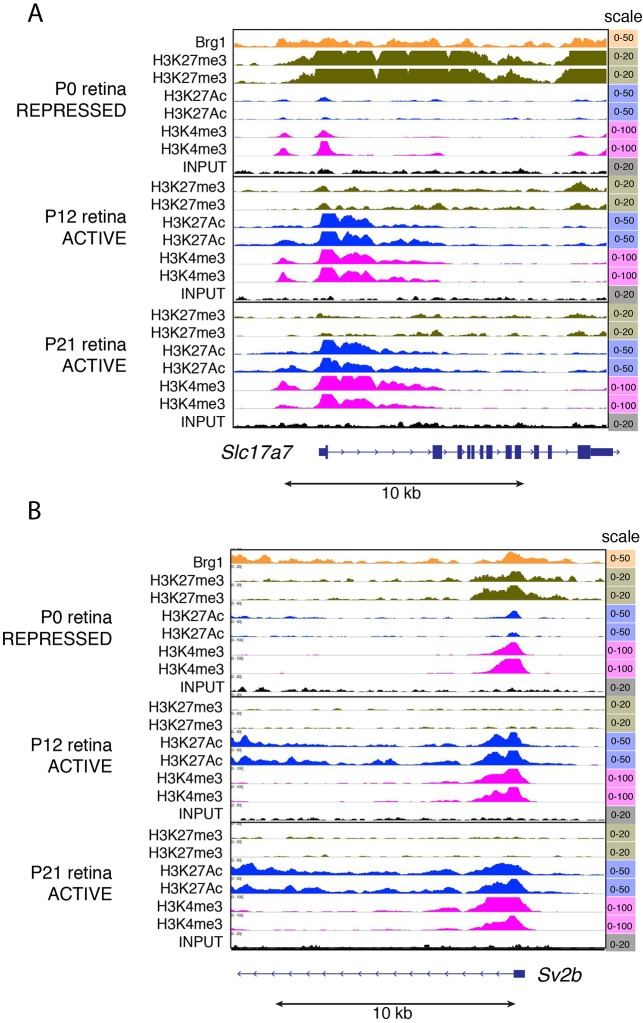
**Brg1-repressed promoters are activated during photoreceptor differentiation.** (A) ChIP-Seq traces for a repressed Brg1 promoter-proximal site in the *Slc17a7* (*VgluT1*) promoter of the P0 retina that is activated at later stages of development (P12-14 and P21). (B) ChIP-Seq traces for a repressed Brg1 promoter-proximal site in the *Sv2b* promoter of the P0 retina that is activated at later stages of development (P12-14 and P21). Both *Slc17a7* and *Sv2b* are downregulated in the P12 and P21 *Brg1*-deficient retinae. Biological duplicate ChIP-Seq is shown for each histone mark at each stage.

To determine if Brg1 binding at P0 influenced gene expression at P14, we determined whether any of the 16,428 promoter-proximal sites at P0 were differentially expressed at P14. We identified 167 Brg1 promoter-proximal genes at P0 that were significantly upregulated (>2-fold difference in gene expression and *P*<0.05) at P14 and identified 62 Brg1 promoter-proximal genes that were significantly downregulated at P14 (Table S9). Pathway analysis using DAVID v6.7 identified neurotransmitter transport as the only significant association (33-fold enrichment; *P*=0.0002, Benjamini-adjusted *P*=0.03) for downregulated genes and this is consistent with the defect in photoreceptor differentiation. Specifically, *Slc17a7* (*VGluT1*) and *Sv2b* are both expressed in photoreceptor terminals and *Rims1* is mutated in cone rod dystrophy type 7 (Phillips et al., 2010; Wang et al., 2003; Johnson et al., 2003; Michaelides et al., 2005). The promoter regions of *Slc17a7* and *Sv2b* where Brg1 binds are repressed at P0 and then become active at P14 and P21 (Fig. 6A,B). These data are consistent with Brg1 playing a role in derepressing the bivalent *Slc17a7* and *Sv2b* promoters during photoreceptor differentiation. However, the *Rims1* promoter is active at P0, P14 and P21 (Fig. S6), so Brg1 might be important for this class of promoters as well. *Pvlr3*, *Cep192* and *Lman1* are similar in epigenetic profiles to *Rims1* (Fig. S7, Fig. 4F). At P0, the *Wif1* and *Mid1* promoters are bivalent (Fig. S8) but, in the absence of Brg1, *Wif1* is upregulated and *Mid1* is downregulated (Fig. 3A,B, Fig. 4A). Acetylation, phosphoproteins and ribosomal genes were the only significantly upregulated pathways in our analysis, with Benjamini-adjusted *P*-values of 3×10^−7^, 4×10^−4^ and 0.001, respectively. There were several retinal genes in these groups, including *Rp9*, *Crabp1* and *Six6.* However, the most striking group of genes in these pathways were associated with the cytoskeleton and cell adhesion (*Tubb2*, *Tubb6*, *Tubb3*, *Mapt*, *Twf2*, *Cfl2*, *Tmsb4x*, *Myl6*, *Arpc3*, *Fnbp1l*, *Cdc42bpg*, *Dynlt1b*, *Vim* and *Rhoj*).

To extend our study, we correlated Brg1 binding at the 33,389 promoter-distal Brg1 sites in P0 retinae with changes in gene expression (within 100 kb) at P12. We identified 46 Brg1 promoter-distal genes at P0 that were significantly upregulated (>2-fold difference in gene expression and *P*<0.05) at P12 and identified 27 Brg1 promoter-proximal genes that were significantly downregulated at P14 (Table S9). There was no significant pathway enrichment for the upregulated or downregulated genes.

It has been shown previously that Brg1 can play a role in nucleosome positioning at promoters (Alexander et al., 2015; Hu et al., 2011) and this may alter gene expression. Alternatively, it is possible that the changes in histone modifications that accompany gene activation or repression during development might be altered in *Brg1-*deficient retinae. To distinguish between these possibilities, we performed ChIP-Seq for H3K4me3, H3K27me3 and H3K27Ac in P12 *Brg1*-deficient retinae and compared them with wild-type retinae. We could not detect any significant differences in the pattern of H3K4me3, H3K27me3 and H3K27Ac in P12 *Brg1*-deficient retinae for the upregulated and downregulated genes. Next, we calculated the distance between the +1 and −1 nucleosomes using the H3K27Ac ChIP-Seq data. There was no significant difference in nucleosome positioning between wild-type and *Brg1*-deficient retinae for the genes that were upregulated, but there was a significant difference for those genes that were downregulated, in *Brg1*-deficient P12 retinae (Table S9). Specifically, using the Wilcoxon signed-rank test (paired), we found that the median +1 to −1 nucleosome distance was 589 bp in the wild-type P12 retina, whereas it was 482 bp (*P*=0.018) and 519 bp (*P*=0.044) in the two biological replicates for *Brg1*-deficient retinae.

### Brg1 is a tumor suppressor in retinoblastoma

**Fig. 7.**
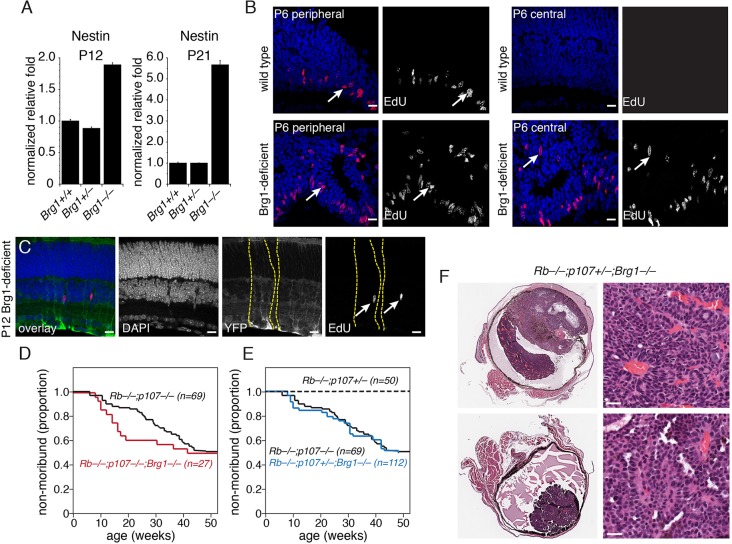
**Brg1 is a tumor suppressor in retinoblastoma.** (A) Quantitative PCR analysis for nestin expression at P12 and P21 using TaqMan probes. Each bar is the mean and s.d. of duplicate PCR reactions in triplicate samples. (B) P6 retinal sections stained for EdU (red) and counterstained with DAPI to label nuclei (blue). Arrows indicate representative immunopositive cells in the central and peripheral retina. (C) P12 *Chx10-Cre;Brg1^Lox/Lox^;Rosa-YFP* retinae. Mosaic regions with Cre-mediated recombination are indicated by green immunofluorescence and EdU^+^ nuclei are indicated by red fluorescence (arrows). Yellow dashed lines indicate the boundaries of the domains where Cre was active and *Brg1* was inactivated. (D,E) Survival curves for genetically engineered mouse models of retinoblastoma with Brg1 conditional inactivation. (F) Retinoblastomas from *Chx10-Cre;Rb^Lox/Lox^;p107^+/−^;Brg1^Lox/Lox^* mice stained with Hematoxylin and Eosin*.* Scale bars: 10 µm in B,C; 25 µm in F.

As described above, conditional inactivation of *Brg1* in the developing mouse retina leads to microphthalmia due to a combination of cell death and lengthening of the cell cycle. Based on these data alone, the inactivation of *Brg1* would be expected to reduce retinoblastoma initiation and/or progression. However, the persistence of immature cells in the postnatal *Brg1*-deficient mouse retina, as seen in electron micrographs and in the expression of progenitor and G2/M genes such as nestin (Fig. 7A), could indicate conditions that have the opposite effect, i.e. of promoting retinoblastoma. Indeed, there are persistent EdU^+^ cells in P6, P12 and P21 *Brg1*-deficient relative to wild-type retinae (Fig. 7B,C; data not shown). To distinguish between these two models of the role of Brg1 in retinoblastoma, we crossed the *Chx10-Cre;Brg1^Lox/Lox^* strain to mice that develop retinoblastoma. First, to determine if inactivation of *Brg1* can prevent tumorigenesis, we compared mice with conditional inactivation of *Rb* (*Rb1*) and *p107* in the developing retina (*Chx10-Cre;Rb^L^^ox/Lox^;p107^−/−^*) with those lacking *Rb*, *p107* and *Brg1* (*Chx10-Cre;Rb^L^^ox/Lox^;p107^−/−^;Brg1^Lox/Lox^*). There was no difference in the proportions of mice that developed retinoblastoma over a 12-month period (Fig. 7D).

Next, to determine if *Brg1* inactivation promotes retinoblastoma tumorigenesis, we generated *Chx10-Cre;Rb^L^^ox/Lox^;p107^+/−^;Brg1^Lox/Lox^* mice and compared tumor formation in these animals with that in *Chx10-Cre;Rb^L^^ox/Lox^;p107^+/−^* and *Chx10-Cre;Rb^L^^ox/Lox^;p107^−/−^* mice. The *Chx10-Cre;Rb^L^^ox/Lox^;p107^+/−^* mice did not develop retinoblastoma during the first year of life. However, the *Chx10-Cre;Rb^L^^ox/Lox^;p107^+/−^* mice lacking *Brg1* developed retinoblastoma at a similar frequency to that seen in *Chx10-Cre;Rb^L^^ox/Lox^;p107^−/−^* mice (Fig. 7E). These results are consistent with the hypothesis that the persistence of retinal progenitor cells in *Brg1*-deficient retinae contributes to retinoblastoma, despite the microphthalmia phenotype.

We also compared the gene expression profiles of the retinoblastomas from *Chx10-Cre;Rb^L^^ox/Lox^;p107^+/−^;Brg1^Lox/Lox^* (*n*=7) with that from *Chx10-Cre;Rb^L^^ox/Lox^;p107^−/−^* (*n*=20) mice. Pathway analysis of the genes expressed in the *Brg1*-deficient tumors identified those involved in mitochondrial homeostasis as the most upregulated, and those involved in neuronal differentiation as the most downregulated (Table S10). Of the 136 upregulated genes 39.7% (54/136) had active marks and 26.4% (36/136) had bivalent marks at P0 in the normal retina (Table S10). For the 111 downregulated genes, 38.7% (43/111) had active marks and 27.9% (31/111) had bivalent marks (Table S10). We also analyzed histone spacing of the retinoblastoma upregulated and downregulated genes using the P12 ChIP-Seq data but there was no statistically significant difference (Table S10). Taken together, these data suggest that Brg1 can act as a tumor suppressor in murine retinoblastoma on a sensitized background (*Chx10-Cre;Rb^L^^ox/Lox^;p107^+/−^*).

## DISCUSSION

Conditional inactivation of *Brg1* in the developing mouse retina reduces the size of the eyes and retinae. The reduced retinal size is caused by a combination of increased cell cycle length, premature cell cycle exit, and increased cell death during retinogenesis. Despite these defects in retinal progenitor cell proliferation during development, retinal progenitor cells persisted in the mature retina. *Brg1* deficiency, when combined with Rb pathway deficiency, also enhances retinoblastoma tumorigenesis, suggesting that Brg1 is a tumor suppressor in the developing murine retina. In addition to the effect of Brg1 loss on proliferation and tumorigenesis, we discovered that Brg1 is required for proper retinal lamination throughout retinogenesis but is dispensable for retinal cell fate specification. Although rod and cone photoreceptors are specified appropriately in the *Brg1*-deficient retinae, their differentiation is perturbed, leading to retinal degeneration. Brg1 binds the promoter region of many of the dysregulated genes involved in cell polarity, proliferation, and photoreceptor differentiation. Together, our data suggest that Brg1 plays a crucial role in coordinating multiple processes during retinal development through cell-autonomous and non-cell-autonomous mechanisms.

### Brg1 loss leads to microphthalmia

Brg1 is an epigenetic regulator that modulates the expression of thousands of genes across the genome in a cell type- and developmental stage-specific manner (Attanasio et al., 2014). Therefore, it can be very difficult to discern direct mechanisms from secondary indirect mechanisms that contribute to developmental phenotypes resulting from deletion of *Brg1* in the developing retina. The most pronounced phenotype in adult *Chx10-Cre;Brg1^Lox/Lox^* mice is microphthalmia. More detailed analyses of embryonic and early postnatal retinae showed that the reduced retinae and eye size were evident early in retinogenesis. It is important to note that the reduction in eye size was not 100% penetrant in our *Chx10-Cre;Brg1^Lox/Lox^* mice because of the mosaic expression of the *Cre* transgene (Fig. S1). The microphthalmia and the disruption in retinal lamination were only observed in retina with high-level expression of the *Cre* transgene, and the majority (∼90%) of cells with evidence of Cre-mediated recombination lack Brg1 protein expression.

Our data suggest that three factors contribute to microphthalmia in *Chx10-Cre;Brg1^Lox/Lox^* mice. First, cell death is slightly increased throughout retinal development. Although this increase is small, loss of even a small number of retinal progenitor cells early in development can significantly affect overall retinal size because individual retinal progenitor cells can produce dozens of postmitotic daughter cells. Second, the proportion of EdU^+^ cells was reduced in *Brg1*-deficient retinae, suggesting that some of the retinal progenitor cells prematurely exited the cell cycle. This is consistent with the reduced expression of retinal progenitor cell genes in the *Brg1*-deficient retina at E14.5. Moreover, in other tissues in which *Brg1* has been inactivated during development, premature cell cycle exit is a major cause of cells having the altered developmental phenotype (Hang et al., 2010; Matsumoto et al., 2006; Wurster and Pazin, 2008). We cannot rule out the possibility that increased cell death contributes to the decreased proportion of EdU^+^ cells, if those dying cells are retinal progenitor cells. The third feature of *Brg1-*deficient retinae that contributes to microphthalmia is increased cell cycle length. An increase in cell cycle length per se would not alter the proportion of EdU^+^ cells if the relative length of the S-phase was maintained in the *Brg1-*deficient retinae. These data highlight how difficult it can be to elucidate the underlying cellular mechanisms of developmental phenotypes that result from perturbations in the SWI/SNF complex.

The *Chx10-Cre* transgene used in this study exhibits mosaic expression and the level of expression can vary from mouse to mouse (Rowan and Cepko, 2004). Therefore, it is likely that the phenotype would be much more severe if a *Cre* transgene were used with broader expression. The extend of *Cre* expression can be monitored using the Rosa-YFP reporter and the expression of human placental alkaline phosphatase from the transgenic allele. The mice with microphthalmia had more extensive Cre expression than those with normal size eyes. We preferentially selected the mice with microphthalmia for the gene expression and ChIP-Seq studies to reduce the contamination of the wild-type retinal cells and we used mice with normal size eyes to study the cell-autonomous effects of *Brg1* inactivation in individual clusters of retinal progenitor cells.

### Brg1 loss leads to retinal degeneration

Despite the reduction in retinal size, we found no significant difference in the proportion of retinal cell types, as measured by immunostaining at P12. However, by P21 the proportion of rods and cones in the *Brg1-*deficient retinae was dramatically reduced. This might have been caused by a defect in retinal differentiation that occurred subsequent to rod and cone cell fate specification; or, it might be a secondary effect of the defects in retinal lamination (see below); or it might be due to a combination of the two processes. To distinguish between these possibilities, we performed gene expression array analysis of wild-type and *Brg1*-deficient retinae at P12 and P21. At P12, prior to the loss of committed photoreceptor cells, the expression of rod and cone photoreceptor genes was already substantially downregulated. Indeed, many of those genes were direct targets of Brg1. The gene lists we used for the different retinal cell types were derived from single-cell gene expression array profiling performed by the Cepko laboratory (Trimarchi et al., 2008, 2007, 2009; Cherry et al., 2009; Roesch et al., 2008, 2012; Mizeracka et al., 2013a,b; Nygaard et al., 2008). Clearly, this is not a comprehensive list of cell type-specific genes. However, it is an unbiased list that was developed through unsupervised clustering of the single-gene expression signatures and many of the genes were validated by *in situ* hybridization.

These data suggest that Brg1 is important for transcriptional activation of the photoreceptor differentiation program in the developing retina. In the absence of Brg1, those genes do not fully activate, photoreceptor differentiation is perturbed, and the committed postmitotic photoreceptors eventually die. The promoters of *Slc17a7*, *Sv2b* and *Lman1* are direct targets of Brg1 and are bivalent at P0. As photoreceptors differentiate, they become activated. We propose that, in the absence of Brg1, derepression is perturbed in photoreceptors. Brg1 might also play a role in activating promoters such as *Rims1* even if they are not bivalent. This proposed cell-intrinsic mechanism of retinal degeneration does not preclude other mechanisms.

Beyond microphthalmia, the most striking histological feature in *Brg1*-deficient retinae was the disruption in cell polarity and retinal lamination. This defect was obvious from E14.5 and persisted throughout development. The photoreceptors that formed were often found in rosettes within the retina, and this most likely contributed to secondary photoreceptor degeneration. Specifically, as photoreceptors differentiate, their outer segments must come into contact with the overlying retinal pigment epithelial cells, which play an essential role in phagocytosis of the photoreceptor outer segments. When those interactions are disrupted, as in the *Brg1*-deficient retinae, the photoreceptors die. Therefore, we propose that the cell-intrinsic defect in photoreceptor differentiation that results from *Brg1* inactivation, combined with the non-cell-autonomous photoreceptor cell loss as a result of defects in retinal lamination, are the major factors that contribute to retinal degeneration in *Chx10-Cre;Brg1^Lox/Lox^* retinae.

### Brg1 and WNT pathway dysregulation

The WNT pathway antagonist *Wif1* is upregulated in *Brg1*-deficient retinae. Wif1, Wnt4, Fzd4 and Lrp6 are expressed in the developing retina and may play a role in rod production and/or survival. Specifically, dissociated P0 retinal cells in culture show decreased rod differentiation in the presence of recombinant Wif1 protein and increased rod production in the presence of Wnt4 or anti-Wif1 antibody (Hunter et al., 2004). These data suggest that, in addition to the cell-autonomous defect in rod differentiation discussed above, a non-cell-autonomous component might be mediated through WNT signaling in the *Brg1*-deficient retinae.

Perturbations in WNT signaling may also contribute to the defect in lamination. Clearly, several of the genes implicated in cell polarity and cell adhesion are direct targets of Brg1, suggesting a cell-autonomous role for Brg1 in cell polarity and retinal lamination. Previous studies have shown that inactivation of *Ctnnb1* leads to defects in retinal lamination that are indistinguishable from the changes that we report here for *Brg1*-deficient retinae (Fu et al., 2006). As shown previously for the *Ctnnb1*-deficient retinae (Fu et al., 2006), we observed defects in localization of N-cadherin, F-actin, aPKC, Par3 and Par6 in the *Brg1*-deficient retinae. The Brg1 ChIP-Seq data suggest that *Wif1* is a direct target. The *Wif1* promoter is bivalent at P0 and remains bivalent throughout retinal development. Our data are consistent with derepression of the *Wif1* promoter during retinal development and this may contribute to defects in WNT signaling during retinogenesis in the *Brg1-*deficient retinae.

### Cross-species comparison of *Brg1* inactivation

The role of Brg1 in retinal development has previously been characterized using the *Brg1* ortholog *yng* (*smarca4a* – ZFIN) in zebrafish (Link et al., 2000; Gregg et al., 2003). The *yng*-deficient zebrafish show a defect in retinal lamination starting at the stage when plexiform layers begin to form (Link et al., 2000; Gregg et al., 2003). Plexiform-like areas form occasionally in the *yng* mutant fish, but the retinae are immature. Later during development, cell death is elevated in the *yng* mutant retinae (Link et al., 2000; Gregg et al., 2003). The initiation and pattern of retinogenesis are normal in the *yng* mutant retinae, but retinal development progresses more slowly. Moreover, retinal cell fate specification is normal in the *yng* mutant retinae, but there are broad defects in differentiation (Link et al., 2000; Gregg et al., 2003).

Most of the zebrafish phenotypes are similar to those seen in *Brg1*-deficient mouse retinae, as we have shown here: reduced eye size, increased cell death, defective retinal lamination, persistence of retinal progenitor cells, and normal cell fate specification. More specific analysis of cell cycle length was not carried out in the *yng* mutant fish, so whether the slowing of retinogenesis in that model is due to increased cell cycle length is unknown.

A major difference between the previous studies on *yng* mutant fish and our analysis of the *Brg1*-deficient mouse retina is the role of the gene in cell differentiation. In *yng* mutant fish, differentiation of most cell types was perturbed, but mosaic analysis suggested that this result was largely due to non-cell-autonomous effects. In our study, the differentiation of photoreceptors was affected, but that of the other cell types was not. Therefore, the cell-autonomous, photoreceptor-specific role of *Brg1* in differentiation may differ across species. Our data also provide insight into the underlying molecular mechanisms that govern the coordination of cell proliferation, specification and differentiation by SWI/SNF complexes in retinal development and retinoblastoma.

### Brg1 is a tumor suppressor in retinoblastoma

The BRG1 subunit of the SWI/SNF complex is mutated in human cancer and has been shown to be a tumor suppressor in mice (Love et al., 2012; Hodis et al., 2012; Parsons et al., 2011; Medina et al., 2008; Shain et al., 2012; Bultman et al., 2000, 2008; Glaros et al., 2008). A homolog of BRG1 called BRM (SMARCA2) can also regulate chromatin structure and is mutually exclusive with BRG1 in the SWI/SNF complex. Importantly, two recent studies have shown that when BRG1 is inactivated there is greater incorporation of BRM into the SWI/SNF complex and this contributes to tumorigenesis (Wilson et al., 2014; Hoffman et al., 2014). Indeed, inactivation of BRM1 is synthetically lethal in BRG1-deficient tumors. Although BRG1 is not mutated in human retinoblastoma, epigenetic dysregulation contributes to tumorigenesis (Zhang et al., 2012). Therefore, we tested the tumor suppressor function of Brg1 in genetically engineered mouse models of retinoblastoma.

Retinoblastoma failed to develop in mice in which *Brg1* was conditionally inactivated, but when *Brg1* inactivation was combined with biallelic inactivation of *Rb1* and *p107* tumorigenesis was accelerated compared with biallelic inactivation alone. The overall proportion of mice that developed retinoblastoma was not affected, only the time to progression. These data indicate that Brg1 inactivation can accelerate retinoblastoma progression. However, one important consideration is the reduction in eye size. The actual rate of tumor initiation and/or progression may be unaltered in the *Chx10-Cre;Brg1^Lox/Lox^;Rb^Lox/Lox^;p107^−/−^* mice, and the apparent acceleration might be due to the smaller eye size. Specifically, the mice reach moribund status more quickly because their eyes are smaller; thus, there is less volume for the tumors to fill before intraocular pressure is elevated. It is also possible that the defects in eye morphogenesis alter vitreal homeostasis, and the intraocular pressure is elevated more readily as tumors begin to grow.

Even if the tumors grow more rapidly in the *Chx10-Cre;Brg1^Lox/Lox^;Rb^Lox/Lox^;p107^−/−^* mice, that does not, in itself, demonstrate that Brg1 is a tumor suppressor in retinoblastoma. More direct evidence that Brg1 is a tumor suppressor in retinoblastoma came from studies of sensitized mice. Specifically, *Chx10-Cre;Rb^Lox/Lox^;p107^+/−^* mice do not develop retinoblastoma, but *Chx10-Cre;Brg1^Lox/Lox^;Rb^Lox/Lox^;p107^+/−^* mice do, with a similar time to progression and incidence as *Chx10-Cre;Rb^Lox/Lox^;p107^−/−^* mice. We were unable to determine if this result was due to genomic instability and inactivation of the second allele of *p107* because it is impossible to isolate tumor tissue of sufficient purity for genomic analyses from individual mice, given the relatively small eye size. This is a reasonable hypothesis considering the downregulation in Cep192, which plays a role in centrosome separation, spindle formation, and centrosome/centriole segregation into daughter cells (Joukov et al., 2014; Firat-Karalar et al., 2014). It is also possible that the other changes in retinal development described above underlie the tumor-suppressor function of *Brg1* in retinoblastoma. For example, the persistent progenitor cells in the P12 *Brg1-*deficient retina might be the source of tumors in *Chx10-Cre;Brg1^Lox/Lox^;Rb^Lox/Lox^;p107^+/−^* mice. Alternatively, perturbations in cell adhesion and cell polarity in the *Brg1*-deficient retinae might contribute to retinoblastoma tumorigenesis. In either case, these data provide additional evidence of the importance of epigenetic processes in retinoblastoma initiation and progression (Zhang et al., 2012).

## MATERIALS AND METHODS

### Animals

*Brg1^Lox/Lox^* mice were obtained from J. Crabtree at Stanford University. *Chx10-Cre;Rb^Lox/Lox^;p107^−/−^* mice were described previously (Ajioka et al., 2007). For tumor formation experiments, mice were monitored weekly for signs of retinoblastoma and anterior chamber invasion. Moribund status was defined as the point when tumor cells invaded the anterior chamber, and intraocular pressure was substantially elevated for at least two sequential readings. The St. Jude Animal Care and Use Committee approved all animal studies. Brg1-FLAG mice were obtained from the Pennacchio laboratory at Lawrence Berkeley National Laboratory, with flash-frozen retinal tissue provided for ChIP-seq analysis.

### Clonal analysis

Procedures for maintaining mouse retina explants in culture were described previously (Dyer and Cepko, 2001b). One retina from each *Brg1^Lox/Lox^* embryo was infected with a retrovirus (NIN) that encodes nuclear β-galactosidase, and the other retina was infected with a retrovirus (NIN-Cre) that encodes nuclear β-galactosidase and Cre recombinase. After 2 weeks in culture, individual retinae were fixed, stained with X-gal and sectioned for scoring.

### EdU and [^3^H]-thymidine labeling

Procedures for immunostaining and EdU labeling were described previously (Martins et al., 2006). Briefly, to label S-phase retinal progenitor cells, we incubated P0 cultured retinal explants with [^3^H]-thymidine (5 µCi/ml, 89 Ci/mmol) for 1 h at 37°C. Cultured retinae were washed and then labeled by addition of 10 mM BrdU (Boehringer Mannheim) at different time intervals. Labeled retinae were dissociated and mounted on slides for immunostaining and then processed for autoradiography. Retinal immunohistochemistry was performed as previously described (Martins et al., 2006). Antibodies and dilutions used in immunostaining retinal sections and dissociated cells are listed in Table S11.

### RNA, qPCR and gene expression array analysis

RNA extraction and qPCR were performed as described previously (Martins et al., 2006). Real-time PCR experiments were performed using the ABI 7900 HT sequence detection system (Applied Biosystems). For microarray analysis, Affymetrix MOE 430 v2 arrays were used. Genes with a minimum mean RMA signal of 6 in one class were preserved. Only annotated genes with a false discovery rate (FDR) of less than 0.05 were included (see the supplementary Materials and Methods). For pathway analysis, we used the DAVID (http://david.abcc.ncifcrf.gov/summary.jsp) version 6.7 toolset. Non-redundant annotated genes with an FDR of less than 0.05 and mean fold difference greater than 2.0 were included in the pathway analysis for each stage. Microarray data are available at GEO under accession no. GSE74181.

### Nuclear shape analysis

For the individual nuclear images shown in Fig. S2, an intensity mask was created to highlight the individual nuclei. The purpose of this modification was to highlight only a particular nucleus in the field. The boundaries of the nucleus were not altered. For scoring, unaltered confocal images were used.

### Immunoblotting

Western blotting was performed as described (Benavente et al., 2014). Anti-PP2Ac (1:4000; Millipore, 05-421) was used. Signals were measured using integrated intensity (counts) detected with an Odyssey infrared imaging system (LI-COR) at 680 and 800 nm.

### Transmission electron microscopy (TEM)

TEM was performed according to the procedure described by Ajioka et al. (2007).

### ChIP-seq

P0 mouse retinae were cross-linked for 10 min in 1% ChIP-seq grade formaldehyde at room temperature. After adding glycine (0.125 M) to stop the crosslinking, retinae were washed and dounced in PBS. Chromatin was sheared using the TruChIP shearing protocol (Covaris). ChIP was performed using the iDeal ChIP-seq Kit (Diagenode). Immunoprecipitated DNA was eluted using the MinElute PCR Purification Kit (Qiagen) and subjected to qPCR analysis and library construction for sequencing. Antibodies used are listed in Table S12. Integrated data analysis is described in detail in the supplementary Materials and Methods. ChIP-seq data are available at GEO under accession no. GSE74268.
